# Unifying pharmacology, systems biology, and regenerative medicine to advance personalized therapies

**DOI:** 10.3389/fphar.2025.1729610

**Published:** 2025-11-20

**Authors:** Albino Martins

**Affiliations:** 1 3B’s Research Group, I3Bs – Research Institute on Biomaterials, Biodegradables and Biomimetics of University of Minho; Headquarters of the European Institute of Excellence on Tissue Engineering and Regenerative Medicine; AvePark - Parque de Ciência e Tecnologia, Guimarães, Portugal; 2 ICVS/3B’s–PT Government Associate Laboratory, Braga/Guimarães, Portugal

**Keywords:** pharmacology, systems biology, regenerative medicine, integrative pharmacology, regenerative pharmacology, personalized medicine

## Introduction

At the nexus of pharmacology, regenerative medicine and systems biology is a new paradigm known as ‘Integrative and Regenerative Pharmacology’ (IRP). By applying the principles of regenerative medicine and the toolkit of cell and molecular biology into drug discovery and therapeutic action, IRP is an essential advancement of pharmacology. Therefore, the integrative approach, the development of novel targeted therapies and the potential for personalized medicine are IRP’s strongest landmarks.

According to George J. Christ and Alex F. Chen, the convergent of three aspects defines the grand challenge for IPR: 1. implementation of integrative pharmacology strategies. These strategies include studies ranging from *in vitro* and *ex vivo* systems to animal models that recapitulate human clinical conditions, all aimed at developing novel pharmacotherapeutics and identifying mechanisms of action (MoA); 2. development of cutting-edge targeted drug delivery systems (DDSs) capable of exerting local treatment. These DDSs can promote healing without side or off-target effects; 3. the previous approaches should be leveraged to develop transformative curative therapeutics. These therapies are able to improve symptomatic relief of target organ disease or pathology as well as to modulate tissue formation and function (Christ and Chen, 2011).

Traditional pharmacology usually focused on developing drugs to reduce symptoms and alter the course of disease, while IRP aims to restore the physiological structure and function of tissues through targeted therapies. Therefore, IRP has the potential to redefine therapeutic landscapes, although it is not yet a substitute for conventional pharmacology. IRP is still a young field that needs strong interdisciplinary cooperation, standardized manufacturing, and clinical validation to realize its transformative potential. Despite significant challenges, there is growing evidence that the field is getting closer to the clinic (Choudhury and Mathur, 2013).

## Conceptual foundations

Integrative pharmacology is the systematic investigation of the interactions between drugs and humans at the molecular, cellular, organ, and system levels (Zhang et al., 2021). In this field, traditional pharmacology is combined with signaling pathways and networks, bioinformatic tools, and omics (transcriptomics, genomics, proteomics, epigenomics, metabolomics and microbiomics). Integrative pharmacology thus aims to: 1. improve our knowledge, diagnosis, and treatment of human diseases and disorders by breaking down the MoA to ‘basic pharmacology’; and 2. facilitate the prediction of possible targets, pathways, and effects that could provide clues for the development of more effective therapeutics (Ma et al., 2020).

**FIGURE 1:**
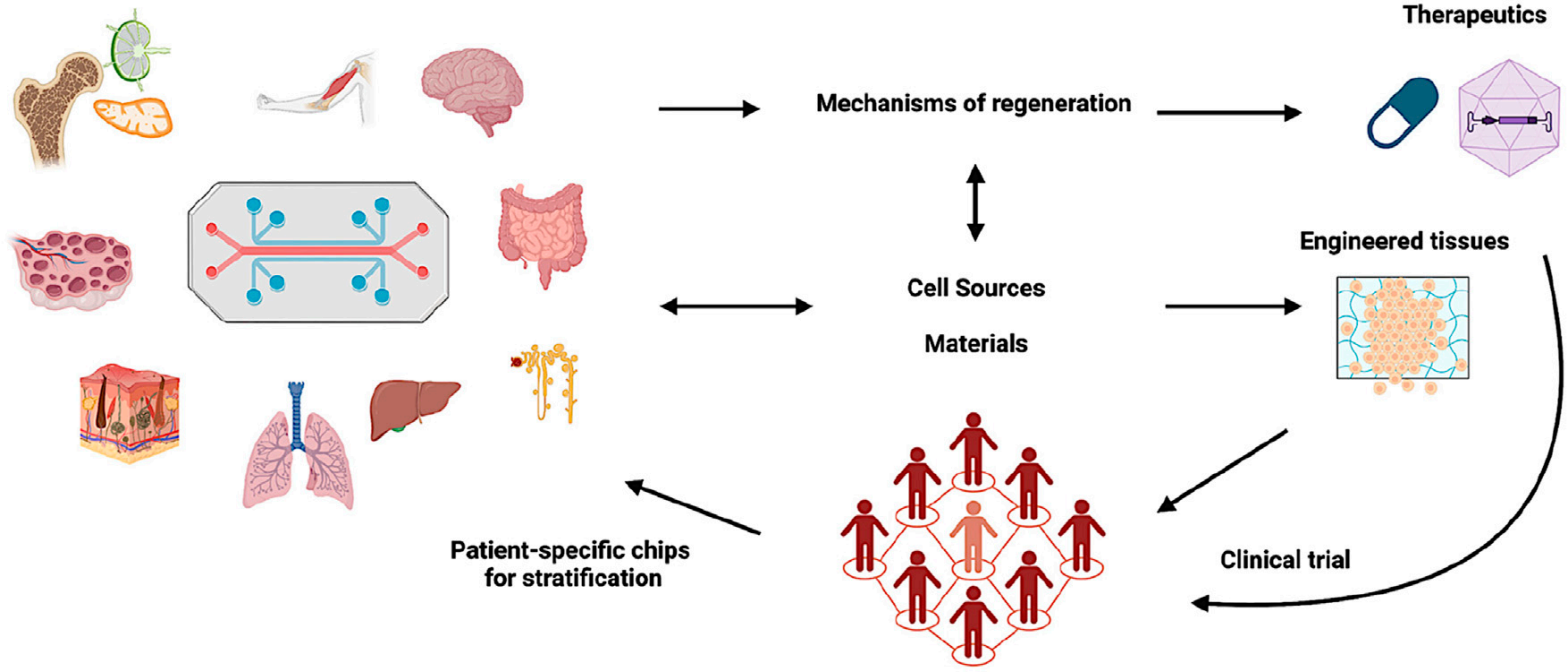
The integrative approach in IRP. Adopted from Goyal et al. (2024).

Alongside, regenerative pharmacology was defined as “*the application of pharmacological sciences to accelerate, optimize, and characterize (either in vitro or in vivo) the development, maturation, and function of bioengineered and regenerating tissues*” (Andersson and Christ, 2007; Christ et al., 2013). This new field is essentially the application of an ancient science to a cutting edge research, fusing pharmacological techniques with regenerative medicine principles to develop therapies that promote the body’s innate healing ability (Williams and Andersson, 2016). Examples of both active (through compounding) and passive (through methodologies) applications of pharmacology in regenerative medicine processes are provided in Table 1. The complementary and synergistic nature of these research areas also permits two-way developments: pharmaceutical innovations can improve the safety and efficacy of regenerative therapies, while regenerative medicine approaches can offer new platforms (e.g., 3D models, organ-on-a-chip) for both drug development and testing (Figure 1; Goyal et al., 2024).

**TABLE 1:** Potential roles of pharmacology in tissue engineering and regenerative medicine (TERM) processes. Adapted from (Andersson and Christ, 2007; Christ et al., 2013).

TERM process	Pharmacological approach
Modulation of cell expansion and differentiation	Use of growth factors, cytokines, hormones or ECM proteins Use of pharmacological methods to characterize cellular phenotype and function
Fabrication of cell/tissue specific functional scaffolds	Biomaterials as cell delivery vehicles or bioactive agents reservoirs for *in vitro* and *in vivo* tissue formation and function
Accelerated maturation of engineered tissues *in vitro*	Use of growth factors, cytokines, hormones or ECM proteins for maximized tissue function Using pharmaceutics in conjunction with other enabling technologies (such as bioreactors) to speed up tissue formation and assessment Pharmacological assessment of tissue development process
Targeted cellular delivery of bioactive agents to modulate regeneration *in vivo*	Development of novel DDS including biomaterials, nanomaterials and biofunctional compounds that target particular tissues Materials sciences to create biocompatible “smart” scaffolds that facilitate engineered tissue integration
Functional evaluation and monitoring of engineered tissues *in vivo*	Integrative and organ systems pharmacology in preclinical studies Integration of pharmacological approaches to imaging and functional assessment modalities

Integrative and regenerative pharmacology is a state-of-the-art interdisciplinary field that bridges pharmacology, systems biology and regenerative medicine, thereby merging the two earlier fields. IRP is the emerging science of restoring biological structure and function through multi-level, holistic interventions that integrate conventional drugs with target therapies intended to repair, renew, and regenerate rather than merely block or inhibit. Therefore, IRP aims to restore rather than just managing pathophysiologic symptoms by introducing the pharmacological rigor into the regenerative space (Christ et al., 2013). IRP is a concept that challenges the traditional drug discovery model and points toward a systems-based, healing-oriented therapeutic approaches, reflecting a paradigm shift in biomedical science. All these theoretical underpinnings position IRP as a field dedicated to both mechanistic rigor and therapeutic innovation.

## Strengths and restraints of IRP

The unifying nature of IRP is its primary strength. It envisions achieving therapeutic outcomes that are not possible with pharmacology or regenerative medicine alone. The IRP represents a paradigm shift in diseases treatment and management by emphasizing both the improvement of tissues’ functional outcomes and the restoration of their structural integrity (Christ et al., 2013). Second, IRP aspires to develop precise therapeutic interventions using genetic profiling and biomarkers of individuals. As part of personalized and precision medicine, state-of-the-art methodologies (e.g., omics, gene editing) are being used to assist in identifying the signaling pathways and biomolecules that are key in the development of novel regenerative therapeutics. The potential of IRP to advance systems pharmacology is another innovation. Modeling of disease networks aids in drug discovery and predicts regenerative pharmacology, allowing for treatments that simultaneously target multiple levels (cells, tissues, organs) of biological organization (Das and Mohideen, 2025). Systems biology methodologies will also help regenerative medicine to define the MoA of therapeutic approaches (e.g., stem cell-derived therapies), accelerating the regulatory approval of advanced therapy medicinal products (ATMPs). Actually, stem cells can be considered as tunable combinatorial drug manufacture and delivery systems, whose products (e.g., secretome) can be adjusted for different clinical applications (Tran and Damaser, 2015).

Despite its promise, IRP faces significant implementation challenges, as evidenced by the numerous preclinical studies but limited number of clinical trials (Williams and Andersson, 2016). Translational barriers rank among the most pressing issues facing IRP advancement, which can be systematized as follows: 1. investigational obstacles, such as unrepresentative preclinical animal models, impact the definition of the therapeutic MoA and raises questions over long-term safety and efficacy; 2. manufacturing issues, such as scalability, automated production methods and technologies, and the need for Good Manufacturing Practice (GMP); 3. complex regulatory pathways with different regional requirements (e.g., EMEA and FDA with no unified guidelines); 4. ethical issues, particularly with regard patients privacy and data security or the use of embryonic stem cell; and 5. economic factors, such as high manufacturing costs and reimbursement (Jacques and Suuronen, 2020). Also, accessibility is ultimately limited by the high cost of ATMPs, especially in low- and middle-income countries. All these uncertainties hamper clinical adoption as well as investment in this emerging field.

## Future perspectives

IRP is a promising field that could be advanced through a number of avenues, including the integration of advanced biomaterials, data-driven approaches through personalized medicine, and the need of expanding clinical trials under collaborative research. Pharmacology and regenerative medicine naturally intersect the biomaterials field (Christ and Andersson, 2013). The development of ‘smart’ biomaterials that can deliver locally bioactive compounds in a temporally controlled manner is expected to be the key of future therapeutics (Christ et al., 2013). Specifically, stimuli-responsive biomaterials, which can alter their mechanical characteristics, shape, or drug release profile in response to external or internal triggers, represent transformative therapeutic approaches. Improved DDSs, such as nanosystems (nanoparticles, nanofibers) and scaffold-based approaches, when combined with imaging capabilities, enable real-time monitoring of physiological response to released compounds or even of the regeneration process (Saul and Harrison, 2013). Despite the constantly evolving role of biomaterials, successful clinical and commercial applications are still lacking. The development of affordable biomaterials and the establishment of standardized, scalable bioprocesses are also crucial for worldwide accessibility.

Artificial intelligence (AI) is a contemporary tool that holds the promise of addressing IRP challenges and improve therapeutic outcomes (Asadi Sarabi et al., 2024). AI has the potential to transform regenerative pharmacology by enabling the development of more efficient and targeted therapeutics, predict DDSs effectiveness as well as anticipate cellular response. A more thorough comprehension of pharmacokinetic and pharmacodynamic profiles in regenerative approaches will be facilitated by developments in omics and gene editing. Utilizing patient-specific cellular or genetic information, advanced therapies can be tailored to maximize effectiveness and minimize side or off-target effects. Challenges remain in implementing AI, namely, the standardization of experimental/clinical datasets and their conversion into accurate and reliable information amenable to further investigation.

Long-term follow-up clinical investigation is required to assess regenerative drugs and biologics beyond initial clinical trials (Choudhury and Mathur, 2013). There is a urgent need to increase the robustness and rigor of clinical trials in regenerative medicine. A reliable validation will require interdisciplinary clinical trial designs that incorporate pharmacology, bioengineering, and medicine. To establish standardized procedures, guarantee consistency in therapeutic outcomes, and eventually develop curative therapies, cooperation between academia, industry, clinics, and regulatory authorities will be essential (Petrosyan et al., 2022). Without this collaborative effort, IRP will not progress from the bench to the bedside, which will specifically affect patients care and the combined ATMPs market segment.

## Concluding remarks

For the time being, the connection between integrative pharmacology and regenerative medicine is essential to bridge the gap between these two fields. More research is required to standardize and scale up manufacturing, fully comprehend the underlying MoA as well as to predict therapeutic outcomes, conduct rigorous clinical investigation and obtain marketing authorization. Every scientific finding made under these stages of development is particularly noteworthy and could contribute to the ‘Integrative and Regenerative Pharmacology’ section of ‘Frontiers in Pharmacology’. In the end, IRP development has the potential to completely transform pharmacology as well as regenerative medicine. The integration of pharmacology, systems biology and regenerative medicine is, therefore, foundational to modern medicine; it is no longer an option. “*Regeneration today must be computationally informed, biologically precise, and translationally agile*” (Ghavami and Los, 2025).
